# Supplementary material for: Empirical methods for controlling false positives and estimating confidence in ChIP-Seq peaks
Source: BMC Bioinformatics. 2008 Dec 5;9:523. doi: 10.1186/1471-2105-9-523 (PMC2628906; doi:10.1186/1471-2105-9-523)
Supplement: Additional File 1 — A variety of html documents from the USeq web site detailing the available applications, their best usage, output file type descriptions, command line menus, etc. [file 1471-2105-9-523-S1.zip › USeqUserGuides/JavaDoc.zip!/JavaDoc/trans/anno/package-frame.html]

trans.anno


trans.anno

Classes 
  
AnnotateRegions
  
AnnotateRegionsWithGeneList
  
BindingRegion
  
BindingRegionComparator
  
BindingRegionsToGff3
  
BindingRegionsToIntervals
  
DmelCGNameCoordinatePrinter
  
FindNeighboringGenes
  
Gff2ToBindingRegions
  
Gff2ToIntervals
  
IntersectBindingPeaks
  
IntersectKeyWithRegions
  
IntersectRegions
  
MergeRegions
  
RandomRegions
  
Region
  
RegionComparator
  
SubtractRegions
